# DXA-Derived Visceral Adipose Tissue (VAT) in Elderly: Percentiles of Reference for Gender and Association with Metabolic Outcomes

**DOI:** 10.3390/life10090163

**Published:** 2020-08-24

**Authors:** Daniele Spadaccini, Simone Perna, Gabriella Peroni, Giuseppe D’Antona, Giancarlo Iannello, Alessandro Faragli, Vittoria Infantino, Antonella Riva, Giovanna Petrangolini, Massimo Negro, Clara Gasparri, Mariangela Rondanelli

**Affiliations:** 1 Endocrinology and Nutrition Unit, Azienda di Servizi alla Persona “Istituto Santa Margherita”, University of Pavia, Via Emilia 12, 27100 Pavia, Italy; gabriella.peroni01@universitadipavia.it (G.P.); clara.gasparri01@universitadipavia.it (C.G.); 2 Department of Biology, College of Science, University of Bahrain, Zallaq 32038, Bahrain; simoneperna@hotmail.it; 3 CRIAMS-Sport Medicine Centre, University of Pavia, 27058 Voghera, Italy; giuseppe.dantona@unipv.it (G.D.); massimo.negro@unipv.it (M.N.); 4 General Management, Azienda di Servizi alla Persona di Pavia, 27100 Pavia, Italy; direttoregenerale@asppavia.it; 5 Department of Internal Medicine and Cardiology, Charité—Universitätsmedizin Berlin, Campus Virchow-Klinikum, Augustenburgerplatz 1, 13353 Berlin, Germany; alessandro.faragli01@gmail.com; 6 Department of Internal Medicine/Cardiology, Deutsches Herzzentrum Berlin, 13353 Berlin, Germany; 7 Berlin Institute of Health (BIH), 10178 Berlin, Germany; 8 DZHK (German Centre for Cardiovascular Research), 10785 Berlin, Germany; 9 Department of Public Health, Experimental and Forensic Medicine, Unit of Human and Clinical Nutrition, University of Pavia, 27100 Pavia, Italy; viriainfantino@hotmail.it (V.I.); mariangela.rondanelli@unipv.it (M.R.); 10 Research and Development Unit, Indena, 20139 Milan, Italy; antonella.riva@indena.com (A.R.); giovanna.petrangolini@indena.com (G.P.); 11 IRCCS Mondino Foundation, 27100 Pavia, Italy

**Keywords:** visceral adipose tissue, diabetes, sarcopenia, kidney, bone

## Abstract

This study aimed to establish the Dual-Energy X-ray Absorptiometry (DXA)-derived Visceral adipose tissue (VAT) reference values for gender and assess the metabolic outcomes associated to the VAT in a cohort of elderly patients. The sample included 795 elderly patients (226/569: men/women) aged 65–100 years (mean age 80.9 ± 7.5ys). Body composition measures and VAT were assessed by DXA and Core-Scan software. Biochemical analysis and a multidimensional comprehensive geriatric assessment were performed. VAT percentiles at the level of 5, 25, 50, 75, 95 were found in males at the following levels: 246, 832, 1251, 1769, 3048 cm^3^ and for females at 99, 476, 775, 1178, 2277 cm^3^. Moreover, this study showed that DXA-VAT was associated to a worsening of lipid, glycemic, hematocrit and kidney profile. Further studies will be needed in order to implement these findings in order to define the (DXA)-derived VAT levels associated to the frailty related risk factors in elderly.

## 1. Introduction

Since 1981, medicine began to accept that some aspects of obesity could also be present in subjects with a normal body mass index (BMI); since then, attention has moved to the analysis of distribution of fat, rather than the actual amount of fat [1]. Intra-abdominal fat accumulation has been recognized to be more associated than subcutaneous fat with insulin resistance and higher cardiometabolic risk [2]. 

Recent studies are confirming that visceral adipose tissue (VAT) is also more responsible for low-grade systemic inflammation compared to subcutaneous adipose tissue (SAT). The physio-pathological mechanisms that explain this aspect are still largely unexplored. However, recent studies report that a clear different behavior between SAT and VAT is mostly evident in visceral obesity. As visceral adiposity progresses, a relatively larger population of immune cells infiltrates VAT; adipocytes become hyperplastic and change their adipokine production, altering the behavior of immune cells and increasing the proinflammatory signaling within the tissue [3,4,5].

Age and gender are the main non-modifiable factors that influence VAT. In both the young and the elderly, men tend to accumulate significantly more fat in the android region compared to women. In addition, VAT increases with age in both genders, but percentual-wise, women show a greater raise of visceral fat, in particular after the menopausal age. In short, body composition in elderly is more similar between genders than younger individuals. On the other hand, older men regularly show greater VAT compared to women, but it is still unclear whether absolute values of VAT are directly associated with proinflammatory cytokines levels and other impairments in the same way in the two genders [6,7].

Moreover, it seems that developmental genes could be responsible of depot-specific differences in adipocyte differentiation and function, making VAT a unique tissue with increased proinflammatory attributes [8]. 

Whatever the cause, free fatty acids, cytokines and adipokines released by VAT are drained by the hepatic portal system causing hepatic alterations and are brought through inferior vena cava, negatively affecting cardiovascular functionality [9].

In general, adipose and muscle tissue linkage is multi-factorial and involves several intertwined aspects. Inflammation is mediated by fat accumulation and muscle inactivity; low physical activity reduces muscle strength and trophism and reduces basal metabolism, promoting weight and fat mass gain; obesity is associated with fat infiltration in muscle and bone tissues [10,11,12,13]. Latest research is also currently confirming that, in elderly, levels of tumor necrosis factor α (TNF-α), interleukin-6 (IL-6) and IL-6/IL-10 ratio are increased in sarcopenic subjects suggesting that VAT may have an important role in muscle atrophy by increasing the production of these cytokines [14,15].

VAT can be decisive in the genesis of sarcopenia and in its various phenotypes related, such as sarcopenic obesity [16,17] and osteosarcopenic obesity [18], to such an extent that the term “osteosarcopenic visceral obesity” was previously defined [19].

Regarding the relationship between VAT and osteoporosis, the literature shows that visceral fat appears negatively associated with bone density, but total body fat and BMI are protective instead. In fact, proinflammatory cytokines mainly produced by VAT increase bone resorption, while adiponectin and leptin can stimulate proliferation and differentiation of osteoblasts, reducing the risk of osteoporosis [20,21,22]. 

Finally, despite the amount of papers describing the relationship between obesity, diabetes, hypertension and renal disease, the direct linkage between visceral adiposity and kidney impairment has not been widely investigated, especially in older adults [23,24]. Few recent studies are reporting that microalbuminuria and lower levels of estimated glomerular filtration rate (eGFR) were detected in patients with increased VAT, but these results were highly dependent on the eGFR formula applied [25,26,27]. 

Given this background, it is clear that VAT has a negative impact on health, but due to its inevitable association with BMI and fat mass, it is still impossible, in a clinical setting, to legitimately evaluate the real impact of VAT on elderly since its absolute values are both associated with protective and threatening effects considering age-related diseases. 

Dual-energy X-ray Absorptiometry (DXA) offers a validated method for studying VAT mass and volume because it offers an accurate estimation together with a lower cost [28].

Regarding the validity of DXA as a direct measurer of VAT, the studies by Neeland et al. and Micklesfield et al. stated that DXA is highly correlated to magnetic resonance imaging (MRI) (R^2^ = 0.82 for females; R^2^ = 0.86 for males) and computed tomography (CT) (R^2^ = 0.957) in the measurement of VAT [29,30]. A recent study by Cheung et al. confirmed this evidence also in elderly subjects. In particular, correlation coefficients were: r = 0.83 for CT and r = 0.902 for MRI [31]. This study aims to assess the association between DXA-derived VAT and several outcomes in a cohort of elderly patients applying different regression models, in order to provide a deep insight on the associations between increased visceral adiposity and older adults health.

## 2. Results

**Figure 1:**
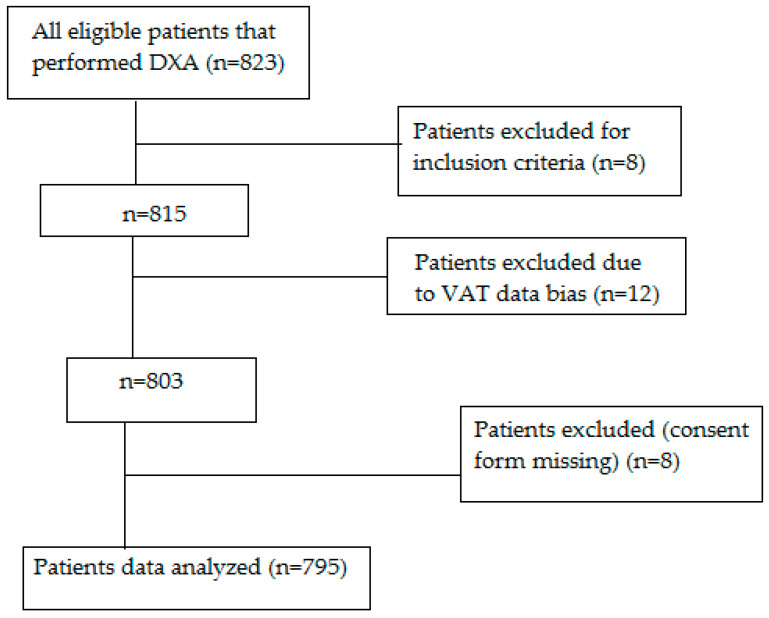
Diagram flow of the study (DXA: Dual-energy X-ray Absorptiometry; VAT: Visceral Adipose Tissue; M: Men; W: Women).

Figure 1 shows the diagram flow of the study. A total of 823 eligible patients performed DXA scan from 2013 to 2018. The final sample obtained by the dedicated Core-Scan application allowed obtaining VAT mass values in 795 subjects (226 men, 569 women, mean age 80.89 ± 7.51 years). 

**Figure 2:**
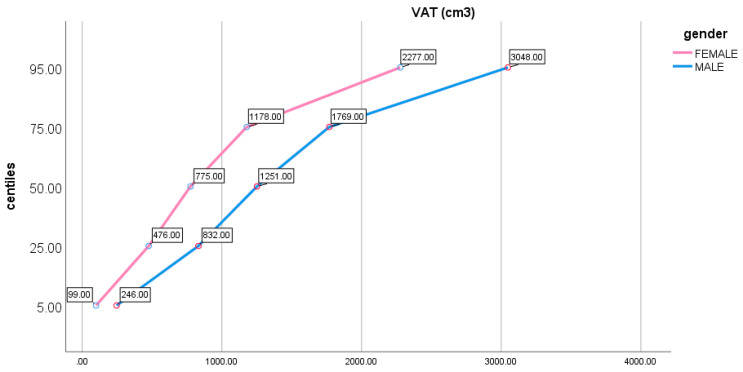
Visceral Adipose Tissue (VAT) average values percentiles in the sample divided for gender.

In Figure 2 are displayed average percentiles values of VAT (expressed in cm^3^) in both genders. A statistically significant difference was found for each level of percentile between the two genders.

**Figure 3:**
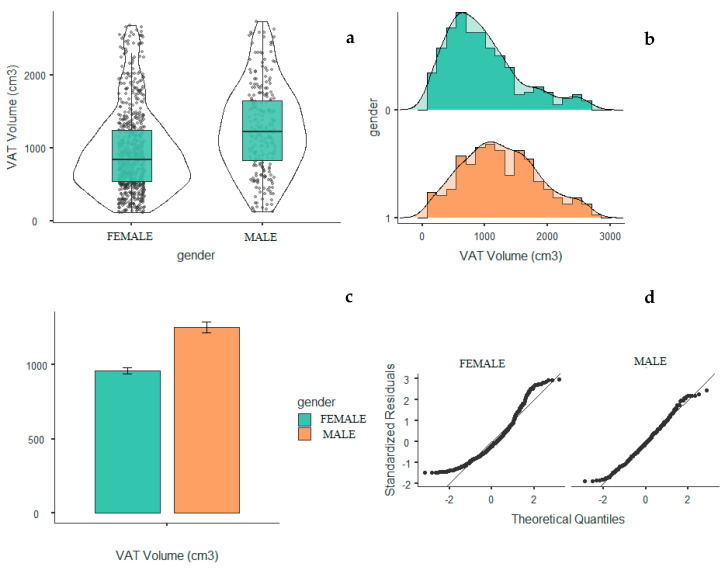
VAT median and mean values in men and women. (**a**)VAT volume median levels for gender (**b**) distribution of VAT valume for genfer (**c**) VAT volume mean levels for gender (**d**) Distribution of theoretical quantiles of VAT for gender.

Figure 3 includes three different views Figure 3a–c of VAT distribution among the sample. Last image Figure 3d expresses how much this distribution fits with the normal distribution in each gender (males are more fitting with normal distribution for VAT variable). 

Supplementary Table S1 shows the baseline characteristics of the sample. Patients had an average BMI of 24.82 ± 4.97 kg/m^2^. Mini nutritional assessment (MNA) mean score was 17.87 ± 3.38 points and 40.4% where at risk of malnutrition (MNA ≤ 17). 

Mini mental state examination (MMSE) mean value was 18.35 ± 7.15 points, with a prevalence of dementia in 50% of subjects (MMSE < 24). Regarding prevalence of main comorbidities, 22.7% of women and 45.9% of men were found to be sarcopenic, 45.0% of women and 22.2% of men were osteoporotic, 19.2% of women and 22.80% of men had diabetes while 65.5% of women and 53.5% of men were having an eGFR ≤ 60 mL/min. Results of statistically significant binary correlations between VAT and the variables are presented in Table 1.

Table 1 showed a positive correlation between VAT with triglycerides, β-1 globulins, uric acid, glycemia, creatinine, eGFR, weight, BMI, femoral neck BMD, FFM, FM and ASM/h^2^ (*p* < 0.01), while the correlations between VAT and HDL cholesterol, age and hip frax were negative (*p* < 0.01). In addition, as well as Hemoglobin, Lymphocytes and Hematocrit were correlated positively with VAT (respectively, *p* < 0.05 and *p* < 0.01)

All statistically significant correlated variables of Table 1 were included in stepwise analysis in order to determine the best fitting regression model. Best predictive variables were found in fat mass, sex, triglycerides, glycemia, creatinine and β-1 globulins (*p* < 0.01; R^2^ = 0.697).

Table 2 shows the results of linear regression analysis by applying this model. No other statistically significant associations were observed. Diabetes (β = 0.054; *p* < 0.05) and eGFR (β = −0.056; *p* < 0.05) were found to be associated with VAT by respectively removing glycemia and creatinine from the model, while HDL cholesterol and uric acid were having a statistically significant association with VAT when removing triglycerides as covariate (*p* < 0.05). Osteoporosis (*p* = ns) and sarcopenia (*p* = ns) were not statistically associated with VAT. Hip FRAX was near to statistical relevance (β = 0.048; *p* = 0.064).

Secondary analysis, performed by excluding all blood test variables from stepwise regression, provided a second model including sex (*p* < 0.01), weight (*p* < 0.01), fat mass (*p* < 0.01), height (*p* < 0.05), BMI (*p* < 0.05) and ASM/h^2^ (*p* < 0.01) as best predicting independent variables (R^2^ = 0.643). Table 3 shows results of linear regression applied for all variables by applying this model. Other statistically significant associations were found between VAT and triglycerides, glycemia, uric acid, HDL cholesterol, β globulins, creatinine and eGFR (*p* < 0.01). Moreover, in this model, VAT was positively associated with diabetes (*p* < 0.01), but not with osteoporosis and sarcopenia (*p* = ns), even when removing ASM/h^2^ from regressors. In addition, FRAX was not statistically significant (*p* = ns). After the adjustments into the regression model, the Hemoglobin, Lymphocytes and Hematocrit were not found associated significantly to VAT.

## 3. Discussion

In the current cross-sectional study, we performed different analyses in order to study VAT from different points of view. In the first one, simple binary correlation was assessed, and this gave a general insight on relationships between absolute values of VAT and all variables studied.

As expected, VAT was associated primarily with BMI, fat mass and multiple other variables linked to an increase of body weight. In addition, VAT was negatively correlated with osteoporosis and positively with handgrip strength and ASM/h^2^. Since no correction for any confounder was made, we believe that this correlation is simply the reflection of the relationship between BMI, bone density and muscle mass [32,33,34]. 

Subsequent analysis gave the most important results of this study. Sex and total fat mass appear to be the two best independent variables linked to VAT, even when correcting for hematochemical parameters, and total fat mass better predicted VAT compared to body weight or BMI. Triglycerides and glycemia, but not uric acid, resulted to be independent parameters associated with VAT. Scientific opinion is currently in agreement with the fact that VAT has a significant effect on insulin resistance development: VAT and homeostatic model assessment-insulin resistance (Homa-IR) appear to be positively linked [35], and both VAT and liver fat are independently and multiplicatively associated with hepatic insulin resistance [36]. The main mechanism that explains this association has been described by Jung et al., who have suggested that the accumulation of visceral fat stores affects insulin metabolism by releasing free fatty acids. In fact, it is believed that the elevated levels of free fatty acids can induce hepatic insulin resistance, particularly by enhancing gluconeogenesis [37]. Diabetes and increased VLDL production are independent consequences of insulin resistance, leading to an overproduction of triglycerides and low levels of HDL [38]. Results of this study show that HDL was inversely associated with VAT but not independently of triglycerides.

Confirming the findings of other studies, VAT mass resulted to be directly associated with uric acid values. Moreover, in this case, this was not true independently of triglycerides.

The same data, in younger cohorts, was described in a recent study performed by Seyed-Sadjadi et al., in which uric acid was positively associated with VAT mass, but not with SAT mass. At the same time, uric acid was also positively associated with plasma glucose [39]. Yamada et al. showed the same results but clearly explained that both visceral fat and ectopic fat accumulation in the liver are significantly associated with hyperuricemia [40]. Another possible mechanism responsible for this finding could be an overproduction of uric acid, which in mice was shown to depend on an increase in the xanthine oxidoreductase activity, produced in adipose tissue. Moreover, VAT and fatty liver are associated with hyperinsulinemia, which reduces uric acid renal excretion [41]. Given this setting, we can explain the increase of uric acid blood levels in visceral obese subjects, but it is not clear why this increase was more dependent on triglycerides, instead of glucose levels.

Hyperinsulinemia may have a role also in creatinine clearance. In fact, both creatinine (positive association) and eGFR (negative association) resulted to be independently associated with VAT. As previously reported, few studies investigated this relationship, in particular in elderly subjects; moreover, despite most studies considering a deleterious effect of VAT on renal function, the results of these studies are conflicting and cystatin C, not creatinine, appeared better associated with VAT [25,26,27]. It is worth noting that, in this study, creatinine was associated with VAT independently of glycemia, triglycerides and anthropometric measures, suggesting that the mechanism behind this evidence may lay in an earlier stage of VAT-induced malignancies [42,43]. 

The most unpredicted result of this study is the positive association between β-globulins and VAT. β-globulins are a group of serum proteins that raise in several and different health condition. Since no correlation was found between VAT and trasferrin, other β-globulins may be linked to VAT accumulation. Further studies are needed in order to investigate this relationship by analyzing singular β-globulins.

Last section of analysis involved a more restricted regression model, applied by excluding hematochemical parameters from the regression. This section was made with the aim of studying VAT from a simple body fat distribution point of view, rather than investigating the metabolic mechanisms linked to it.

In these terms, independently of sex, weight, BMI, height and fat mass, ASM/h^2^ resulted to be strongly and negatively associated with VAT. Moreover, FFM was negatively associated with VAT, excluding ASM/h^2^, but the latter better estimates this tendence. Handgrip strength was not associated with VAT in this model, neither in the previous model; for this reason, VAT resulted to be not associated with sarcopenia, following the EWGSOP2 criteria.

We are giving the evidence that visceral fat accumulation is associated with a reduction of appendicular muscle mass only in terms of body composition, but this was not independent of metabolic interactions within this tissue. From another point of view, skeletal muscle mass/visceral fat ratio could be a good predictive index of metabolic alterations, as observed in several studies [44,45].

On the other hand, VAT did not show any association with femoral BMD or with osteoporosis. Recent cross-sectional studies reported that an inverse association exists between VAT and BMD [20,46], but this evidence has not been widely accepted; further investigation, including longitudinal perspective studies, has claimed that fracture risk, not BMD, could be the real distinction element between visceral obese/sarcopenic obese subjects and other phenotypes [47,48,49,50]. This may due to the fact that visceral obese subjects tend to have less appendicular muscle mass, increasing the risk of fall; moreover, increased VAT is associated with a modification of bone architecture, especially when combined with high BMI. In fact, obese subjects tend to have increased trabecular BMD and reduced cortical BMD, even if total BMD remains the same [47,49]. For this reason, latest research is currently aiming to evaluate osteoporosis not only in terms of quantity, but also quality. In recent years, trabecular bone score (TBS) was implemented as a novel DXA-based technique for detecting bone microarchitecture and encouraging evidence has already been brought in terms of correlation with BMD, but not with BMI, making TBS an ideal indicator for estimating fracture risk in obese individuals [51]. 

Analysis of percentiles shows that in elderly VAT is significantly different among genders for each level of percentile. In the literature, it is being reported that VAT has a different impact on cardiovascular disease between genders, with a greater negative influence for men and reduced for women, for whom insulin resistance has a predominating role in the cardiovascular disease genesis. Despite this evidence, there are still few studies that investigate on the different impact of VAT in elderly between genders. Nevertheless, the analysis of percentiles provides a preliminary insight of critical amounts of VAT; in fact, similar percentiles could be related to similar outcomes in the two genders [6,7,52]. 

Interesting was the relevance of Hemoglobin and Hematocrit into the context of VAT. In the first step of analysis (correlation) the hemocrome markers resulted associated positively to the VAT. This data was not supported by any scientific report. The most interesting data regarding the hemocrome markers come up from the regression models. The hematrocrit was inversely associated to VAT as demonstrated by the study by Fahrangi 2013. In fact, the literature preliminary showed that hemoglobin, lymphocytes and hematocrit concentrations be added to the cluster of variables related to insulin resistance in patients with visceral fat due to the inflammatory role [53,54].

Taking into account the limitations of this study, the evidence produced, despite the large sample, only applies to the elderly, and in particular only to a subpopulation of older adults who already present some sort of functional loss secondary to a non-disabling condition. Furthermore, even though the findings of this cross-sectional study are preliminary, we believe that few missing data could have been fundamental in order to provide a clearer analysis of potential confounders. As previously said, in fact, the concentration of pro-inflammatory cytokines and free fatty acids might be the real first genesis of VAT-induced derangements; for this reason, further research including a deep analysis of cytokines could definitely solve the disputes implicated in this study. Furthermore, blood pressure and cystatine-C levels, which are key elements for studying kidney disease, were not available. 

## 4. Materials and Methods

### 4.1. Setting

The study was performed at the Santa Margherita rehab clinic (University of Pavia), in the city of Pavia, Italy. 

### 4.2. Study Population

Eligible patients were aged from 65 to 100 years. Data were collected from January 2013 to the end of January 2018. The study design was approved by the ethics committee of the University of Pavia (approval code: 0722/14122918) and an individual written informed consent was obtained from each participant.

Inclusion criteria were (1) admission to the post-acute geriatric care unit for functional loss secondary to a nondisabling medical disease, (2) aged 65 years or older, (3) bedridden patients who were ambulatory prior to hospitalization, and (4) willingness to participate and to provide signed informed consent. At time of admission, the patients were not diagnosed with disabling diseases that could directly affect muscle weakness (such as neurological diseases, hip fractures or amputations). 

Exclusion criteria were subjects affected by acute illness, severe liver, heart or kidney dysfunction or severe dementia. Participants with diabetes, metabolic disease or neoplasia, as well as patients treated with steroids or hormones (except vitamin D) therapies were excluded.

### 4.3. Observed Variables

#### 4.3.1. Body Composition Assessment and Diagnosis of Visceral Adipose Tissue (VAT)

Body composition parameters such as Fat Free Mass (FFM), Fat mass (FM), appendicular skeletal muscle mass (ASM), relative ASM index (ASM/h^2^) and visceral fat data (expressed in grams) were obtained with the use of Dual Energy X-Ray Absorptiometry (DXA) (Lunar Prodigy DXA; GE Healthcare Medical Systems) together with the DXA Prodigy enCORE software (version 17; GE Healthcare). Visceral adipose tissue volume was estimated using a constant correction factor (0.94 g/cm^3^). The software automatically places a quadrilateral box, which represents the android region, outlined by the iliac crest and with a superior height equivalent to 20% of the distance from the top of the iliac crest to the base of the skull [55].

#### 4.3.2. Anthropometric Data Assessment

Body weight: the weight was measured using a calibrated scale. The patient was weighed wearing light clothes (underwear) and without shoes. 

Body mass index (BMI): BMI was calculated as the ratio between body weight and the square of height in meters. The patient is classified as underweight if BMI < 18.5, normal weight if BMI is from 18.5 to 24.9, overweight if BMI is from 25 to 29.9, suffering from obesity of first degree if BMI is from 30 to 34.9, suffering from obesity of II grade if BMI is from 35 to 39.9 and suffering from obesity of III grade if BMI ≥ 40 [54]. Despite this classification, in older subjects, optimal BMI is between 25 and 27/27.5 kg/m^2^, and underweight threshold should be set at 20 kg/m^2^ [56,57,58,59].

#### 4.3.3. Diagnosis of Sarcopenia

Muscle strength has been evaluated through handgrip strength, using a Jamar dynamometer and adhering to the standard procedures recommended by the 2nd edition of American Society of Hand Therapists [60]. The instrument was set at the second handle position from the inside for all testing. Participants were seated with their shoulders adducted and neutrally rotated, elbow flexed at 90°, fore-arm in neutral position and wrist between 0° and 30° of flexion and between 0° and 15° of ulnar deviation [61]. Handgrip strength values were determined as the best values of six measurements (three for each hand). Appendicular Skeletal muscle mass (ASM) was defined as the sum of the lean mass of the arms and the lean mass of the legs [62]. According to the recently published EWGSOP2 consensus on definition and diagnosis of sarcopenia, sarcopenic subjects were having handgrip < 27 kg for men/< 16 kg for women and ASM < 20 kg in men/< 15 kg in women or ASM/height^2^ < 7.0 kg/m^2^ for men/< 5.5 kg/m^2^ for women [63]. Sarcopenia severity was not investigated.

#### 4.3.4. Assessment of Bone Mineral Density 

Bone Mineral Density (BMD) (g/cm^2^) of femoral neck was measured using DXA. BMD is labeled as normal when T- score is >−1.0; we can define osteopenia if T-score is ≤−1.0 and >−2.5 and osteoporosis when T-score ≤−2.5. In addition, we evaluated hip fracture risk assessment tool (FRAX) index that considers femoral neck bone mineral density (BMD) and other osteoporosis risk factors to calculate the fracture risk at 10 years [64]. Lumbar Spine BMD was not considered in the analysis of osteoporosis due to multiple frequent aspects that may increase BMD despite the osteoporosis status (bone fractures, artifacts, compressions, etc.)

#### 4.3.5. Blood Sample Measurements

Fasting venous blood samples were drawn between 8 am and 10 am, with the subjects in a sitting position. Blood handling and collection were carried out under strictly standardized conditions. 

Routine blood tests were performed: hemoglobin concentration (Hb), hematocrit (Hct) and platelet count (PLT) were analyzed in one central laboratory. Serum albumin was also analyzed using a nephelometric method, with a 2% coefficient of variation. Fasting blood total cholesterol and triglycerides levels were measured by automatic biochemical analyzer. High-sensitivity C-reactive protein (CRP), erythrocyte sedimentation rate (ESR), creatinine, glycemia and complete blood count were also assessed. Estimated glomerular filtration rate (eGFR) was calculated through the Cockroft–Gault formula [65]. Patients with fasting plasma glucose ≥ 126 mg/dL or treated with hypoglycemic agents were labeled as diabetic. 

#### 4.3.6. Screening of Cognitive Status

The Mini Mental State Examination (MMSE) is a well-validated and widely used screening tool of global cognitive function. It takes approximately 10 to 15 min to administer and has a maximum score of 30 points. A MMSE < 24 is the conventional cut-off for dementia [66]. 

#### 4.3.7. Assessment of Nutritional Status: (MNA)

The Mini nutritional assessment has been assessed (MNA). MNA comprises 18 questions from four categories: anthropometric assessment, general state, dietary assessment and self-assessment. a maximum of 30 points can be achieved. A score of ≥24 points describes a well-nourished status. A score of 17 to 23.5 points indicates a risk of malnutrition, while less than 17 points indicates malnutrition [67].

### 4.4. Statistical Analysis

All analyses were performed using Statistical Package for the Social Sciences, Version 25.0 (SPSS Inc., Chicago, IL, USA). After verification of the normal distribution of continuous variables, data were statistically analyzed as descriptive statistics. Descriptive statistics representing raw data for each gender and for the full sample were provided, including means and standard deviations. *p*-value between genders was also studied for all variables.

Before applying regressions, binary analysis was performed between VAT and all variables. Given the statistically significant correlations, the best fitting regression model, including the highest number of independent variables, was created from stepwise linear regression analysis. Given the best regression model, all other independent variables were studied through linear regression by applying this model. A secondary analysis was performed by excluding blood test from stepwise linear regression in order to study best fitting model restricted to body composition. Standardized Beta (β) is defined as the standardized increase (or decrease) of a dependent variable (VAT related) for one unit of increase of the independent variable; the significant associations between VAT and the variables are ascertained through this β with a *p*-value < 0.05. Two different significance levels, stated at *p* < 0.05 and *p* < 0.01, were considered. R^2^, which explains how close the data are to the fitted regression model, was displayed for the supreme model. Confidence interval (CI) was always set at 95%.

## 5. Conclusions

In conclusion, this study creates a connection between latest research and a wider perspective of VAT as a source of multiple deleterious outcomes in the elderly. VAT was associated with diabetes, with a worsening of lipid profile and with other metabolic impairments. Moreover, results of this study provide evidence that VAT is negatively associated with kidney function and appendicular muscle mass, even if the association with sarcopenia was not statistically significant. No relationship was found between VAT and osteoporosis. Further cross-sectional studies are required to examine the association of VAT with these diseases including the mediator role of proinflammatory cytokines.

## Figures and Tables

**Table 1:** Statistically significant correlations with VAT.

Variable	r	Variable	r
Gender	0.30 **	Weight	0.72 **
Age	−0.11 **	BMI	0.63 **
White blood cells	0.07 *	MNA	0.33 **
Hemoglobin	0.10 **	Femoral Neck BMD	0.37 **
Hematocrit	0.08 *	Femoral Neck T-score	0.34 **
Lymphocytes %	0.09 *	Hip FRAX	−0.24 **
Triglycerides	0.24 **	FFM	0.38 **
HDL cholesterol	−0.22 **	FM	0.67 **
β Globulins	0.16 **	Handgrip	0.21 **
Creatinine	0.21 **	ASM	0.30 **
eGFR	0.29 **	ASM/h^2^	0.20 **
Amylase	−0.08 *	FFM%	−0.52 **
Uric acid	0.20 **	FM%	0.52 **
Glycemia	0.15 **	Osteoporosis (N = 0/Y = 1)	−0.25 **
Height	0.29 **	Diabetes (N = 0/Y = 1)	0.18 **

BMI: Body Mass Index; MNA: Mini Nutritional Assessment; BMD: Bone Mineral Density; FRAX: Fracture Risk Assessment Tool; FFM: Fat Free mass; FM: Fat Mass; eGFR: Estimated Glomerular Filtration Rate; ASM: Appendicular Skeletal Muscle Mass *: *p* < 0.05; **: *p* < 0.01. Statistically significant correlation between VAT and variable.

**Table 2:** Linear regression model 1. Associations between VAT and other variables.

Variable	β	Confidence Interval 95%	Variable	β	Confidence Interval 95%
Gender (F = 0/M = 1)	0.423 **	595.06; 741.79	Fe (µg/dL)	−0.018	−1.42; 0.63
Age (years)	0.010	−3.89; 6.17	Creatinine (mg/dL)	0.066 **	27.66; 156.41
Height (cm)	0.066	−8.92; 0.29	eGFR (mL/min)	−0.056 ^δ.^*	−3.60; −0.21
Weight (kg)	0.047	−3.04; 8.13	Amylase (U/L)	0.002	−1.29; 1.41
BMI (kg/m^2^)	0.062	−3.93; 22.55	Glycemia (mg/dL)	0.063 **	0.33; 1.94
MMSE (pts)	−0.029	−8.71; 2.61	Uric Acid (mg/dL)	0.052 ^π.^*	1.84; 34.23
MNA (pts)	0.012	−9.17; 14.27	ESR (mm/hr)	−0.035	−1.95; 0.30
White Blood Cells (K/uL)	−0.026	−16.40; 4.53	CRP (mg/dL)	−0.009	−14.08; 9.58
Red Blood Cells (M/uL)	−0.031	−82.16; 17.18	AST (U/L)	0.014	−1.48; 2.86
Hemoglobin (g/dL)	−0.011	−24.80; 14.99	ALT (U/L)	0.004	−1.75; 2.07
Hematocrit (%)	−0.025	−10.73; 3.21	γGT (U/L)	0.027	−0.33; 1.28
Platelets (K/uL)	−0.010	−0.43; 0.27	FM (g)	0.700 **	0.05; 0.05
Lymphocytes (%)	0.035	−0.00; 0.00	FM%	−0.047	−11.02; 5.19
Total Cholesterol (mg/dL)	−0.040	−1.46; 0.15	FFM (g)	0.006	−0.01; 0.01
LDL Cholesterol (mg/dL)	−0.033	−1.60; 0.27	FFM %	0.047	−5.20; 11.02
HDL Cholesterol (mg/dL)	−0.043 ^π,^*	−4.25; −0.16	T-score (sd)	−0.002	−28.09; 25.54
Triglycerides (mg/dL)	0.101 **	0.73; 1.94	Hip FRAX (%)	0.048	−0.34; 10.24
Total blood proteins (g/dL)	−0.040	−96.81; 5.15	ASM/h^2^	−0.005	−31.58; 25.57
Albumin (g/dL)	−0.013	−88.84; 50.85	Handgrip (kg)	−0.027	−8.96; 2.90
α 1 Globulin (%)	−0.007	−26.65; 19.93	Osteoporosis (N = 0/Y = 1)	0.003	−66,88; 74.30
α 2 Globulin (%)	−0.029	−24.27; 5.38	Diabetes (N = 0/Y = 1)	0.054 ^Ω.^*	16.27; 178.28
β Globulins (%)	0.065 **	7.34; 40.07	Sarcopenia (N = 0/Y = 1)	0.000	−73.01; 71.59

BMI: Body Mass Index; MMSE: Mini Mental State Examination; MNA: Mini Nutritional Assessment; eGFR: Estimated Glomerular Filtration Rate; ESR: Erythrocyte sedimentation rate; CRP: C Reactive Protein; AST: Aspartate Aminotransferase; ALT: Alanine Aminotransferase; γGT: Gamma-glutamyl transferase; FFM: Fat Free mass; FM: Fat Mass; FRAX: Fracture Risk Assessment Tool; ASM: Appendicular Skeletal Muscle Mass. *: *p* < 0.05; **: *p* < 0.01. Statistically significant association between VAT and variable; ^π^: triglycerides regressor removed; ^δ^: creatinine regressor removed; ^Ω^: glycemia regressor removed.

**Table 3:** Linear regression model 2. Associations between VAT and other variables.

Variable	β	Confidence Interval 95%	Variable	β	Confidence Interval 95%
Gender (F = 0/M = 1)	0.432 **	577.79; 783.44	Fe (µg/dL)	−0.034	−1.75; 0.23
Age (years)	0.006	−4.16; 5.58	Creatinine (mg/dL)	0.082 **	49.75; 172.03
Height (cm)	−0.635 *	−68.12; −31.87	eGFR (mL/min)	−0.085 **	−4.61; −1.16
Weight (kg)	1.260 **	44.06; 90.43	Amylase (U/L)	0.003	−1.22; 1.41
BMI (kg/m^2^)	−0.797 *	−170.84; −61.55	Glycemia (mg/dL)	0.084 **	0.74; 2.31
MMSE (pts)	0.160	−9.46; 1.56	Uric Acid (mg/dL)	0.081 **	12.75; 43.13
MNA (pts)	−0.026	−16.96; 6.08	ESR (mm/hr)	0.011	−0.81; 1.34
White Blood Cells (K/uL)	0.001	−10.02; 10.34	CRP (mg/dL)	0.012	−8.56; 14.61
Red Blood Cells (M/uL)	−0.036	−85.46; 8.04	AST (U/L)	0.011	−1.61; 2.65
Hemoglobin (g/dL)	−0.033	−32.38; 4.71	ALT (U/L)	0.020	−1.00; 2.77
Hematocrit (%)	−0.033	−11.34; 1.80	γGT (U/L)	0.039	−1.00; 1.51
Platelets (K/uL)	0.018	−0.20; 0.47	FM (g)	0.492 **	0.03; 0.05
Lymphocytes (%)	0.015	−2.24; 4.50	FM%	0.118 ^Σ^	−2.85; 17.40
Total Cholesterol (mg/dL)	−0.004	−0.81; 0.68	FFM (g)	−0.050 *^,Σ^	−0.03; −0.01
LDL Cholesterol (mg/dL)	−0.020	−1.29; 0.51	FFM %	−0.118 ^Σ^	−17.40; 2.85
HDL Cholesterol (mg/dL)	−0.065 **	−5.16; −0.88	T-score (sd)	−0.004	−27.89; 23.56
Triglycerides (mg/dL)	0.127 **	1.05; 2.19	Hip FRAX (%)	0.029	−2.22; 7.71
Total blood proteins (g/dL)	−0.025	−75.35; 20.91	ASM/h^2^	−0.171 **^,Σ^	−137.46; −57.17
Albumin (g/dL)	−0.029	−104.97; 24.24	Handgrip (kg)	−0.040 ^Σ^	−10.68; 1.52
α 1 Globulin (%)	0.024	−10.35; 34.71	Osteoporosis (N=0/Y=1)	0.009	−55.31; 82.51
α 2 Globulin (%)	0.012	−10.68; 18.40	Diabetes (N = 0/Y = 1)	0.068 **	43.33; 201.80
β Globulins (%)	0.061 **	5.98; 37.45	Sarcopenia (N = 0/Y = 1)	−0.045 ^Σ^	−8.24; 147.46

BMI: Body Mass Index; MMSE: Mini Mental State Examination; MNA: Mini Nutritional Assessment; eGFR: Estimated Glomerular Filtration Rate; ESR: Erythrocyte sedimentation rate; CRP: C Reactive Protein; AST: Aspartate Aminotransferase; ALT: Alanine Aminotransferase; γGT: Gamma-glutamyl transferase; FFM: Fat Free mass; FM: Fat Mass; FRAX: Fracture Risk Assessment Tool; ASM: Appendicular Skeletal Muscle Mass. *: *p* < 0.05; **: *p* < 0.01. Statistically Significant association between VAT and variable; ^Σ^: ASM/h^2^ regressor removed.

## Data Availability

The datasets analyzed during the current study are not publicly available due to patients’ privacy but are available from the corresponding author on reasonable request.

## Supplementary Materials

The following are available online at https://www.mdpi.com/2075-1729/10/9/163/s1, Table S1: Descriptive characteristics of the sample.
