# Supplementary material for: DXA-Derived Visceral Adipose Tissue (VAT) in Elderly: Percentiles of Reference for Gender and Association with Metabolic Outcomes
Source: Life (Basel). 2020 Aug 24;10(9):163. doi: 10.3390/life10090163 (PMC7554982; doi:10.3390/life10090163)
Supplement: Supplementary file 1 [file life-10-00163-s001.pdf]

Supplementary

Table S1. Descriptive characteristics of sample.

Variable	Men, Mean ± SD	Women, Mean ± SD	Total, Mean ± SD
**Gender (M/F)**	**226 (n)**	**569 (n)**	**795 (n)**
Age (years)*	79.58 ± 7.52	81.42 ± 7.51	80.89 ± 7.51
MMSE (points)	18.76 ± 7.28	18.18 ± 7.09	18.35 ± 7.15
White Blood Cells (K/uL)	7.35 ± 2.52	6.99 ± 3.24	7.10 ± 3.05
Red Blood Cells (M/uL)	4.21 ± 0.62	4.19 ± 0.69	4.19 ± 0.67
Haemoglobin (g/dL)*	12.63 ± 1.85	12.26 ± 1.64	12.37 ± 1.71
Hematocrite (%)*	38.43 ± 5.59	37.53 ± 4.71	37.79 ± 4.99
PLT (K/uL)*	230.50 ± 83.90	249.70 ± 99.83	244.07 ± 95.78
Limphocytes (%)	24.41 ± 8.17	27.00 ± 10.14	26.26 ± 9.68
Fe (µg/dL)	65.27 ± 34.66	65.93 ± 32.03	65.74 ± 32.78
Transferrin (mg/dL)*	204.46 ± 52.93	233.41 ± 55.82	224.78 ± 56.50
Triglycerides (mg/dL)*	110.54 ± 45.74	123.33 ± 59.19	119.63 ± 55.91
Total Cholesterol (mg/dL)*	167.39 ± 39.68	190.43 ± 43.76	183.79 ± 43.86
LDL Cholesterol (mg/dL)*	102.09 ± 34.68	116.86 ± 36.39	112.48 ± 36.49
HDL Cholesterol (mg/dL)*	43.79 ± 14.27	50.54 ± 15.24	48.61 ± 15.27
Albumin (g/dL)	3.85 ± 4.15	3.63 ± 0.47	3.69 ± 2.25
Creatinin (mg/dL)	1.13 ± 0.71	0.950 ± 2.29	1.00 ± 1.97
eGFR (ml/min)*	58.58 ± 23.89	51.78 ± 19.61	53.74 ± 21.14
Amylase (U/L)	36.51 ± 29.10	35.14 ± 25.73	35.54 ± 26.74
Uric Acid (mg/dL)*	5.47 ± 1.77	5.123 ± 2.14	5.22 ± 2.05
AST (U/L)	19.84 ± 14.95	20.25 ± 13.85	20.13 ± 14.16
ALT (U/L)	18.76 ± 18.90	17.74 ± 15.09	18.04 ± 16.27
γGT (U/L)	33.47 ± 32.25	32.37 ± 40.77	32.69 ± 38.52
Glycaemia (mg/dL)	111.29 ± 43.03	106.40 ± 40.18	107.82 ± 41.06
ESR (mm/hr)	42.40 ± 31.33	44.31 ± 30.03	43.76 ± 30.41
CRP (mg/dL)	4.26 ± 36.36	1.16 ± 2.40	2.05 ± 19.63
Height (cm)*	165.54 ± 7.99	152.91 ± 6.91	156.55 ± 9.22

Weight (kg) *	67.50 ± 12.30	58.33 ± 13.44	60.97 ± 13.75
BMI (kg/m)	24.61 ± 4.02	24.91 ± 5.31	24.82 ± 4.97
MNA (points)	18.25 ± 3.31	17.71 ± 3.40	17.87 ± 3.38
Handgrip (kg)*	21.75 ± 6.56	14.68 ± 5.15	16.71 ± 6.44
ASM (g)	20,021.41 ± 3655.08	14,673.65 ± 3363.66	16,183.84 ± 4204.61
ASM/h² (kg/m²)*	7.31 ± 1.25	6.35 ± 1.17	6.62 ± 1.27
Femoral Neck T-Score (sd)*	−1.48 ± 1.44	−2.39 ± 1.26	−2.13 ± 1.38
Hip FRAX (%)*	2.90 ± 2.50	8.52 ± 8.23	6.90 ± 7.51
FM (g)*	17,602.17 ± 8428.08	20,801.51 ± 10,415.86	19,877.87 ± 9983.99
FM (%)*	25.22 ± 9.09	34.68 ± 11.57	32.14 ± 11.63
FFM (g)*	47,307.44 ± 6645.58	35,787.45 ± 4884.70	39,109.16 ± 7545.43
FFM (%)*	74.22 ± 9.09	65.31 ± 11.57	67,86 ± 11.63
VAT (g)*	1399.32 ± 840.71	902.04 ± 617.97	1045.28 ± 724.90

*: statistically significant difference between genders ($p < 0.05$).
